# Ocular toxoplasmosis during frontline venetoclax therapy for chronic lymphocytic leukaemia – more than meets the eye

**DOI:** 10.1002/jha2.671

**Published:** 2023-03-08

**Authors:** Rory Bennett, Lyndell L. Lim, Constantine S. Tam

**Affiliations:** ^1^ Department of Clinical Haematology Peter MacCallum Cancer Centre Melbourne Australia; ^2^ Victoria Parade Eye Consultants Fitzroy Australia; ^3^ Centre for Eye Research Australia University of Melbourne Melbourne Australia; ^4^ Department of Haematology The Alfred Hospital Melbourne Australia; ^5^ Australian Centre for Blood Diseases Monash University Melbourne Australia

1

A 67‐year‐old woman presented with a right central scotoma and reduced Snellen visual acuity (<6/60), 4 months after commencing frontline obinutuzumab–venetoclax (Ven–O) therapy for 13q‐deleted, IGHV‐mutated, chronic lymphocytic leukaemia (CLL). At the time of ocular symptoms, her full blood count comprised haemoglobin 131 g/L, lymphocytes 0.7 × 10^9^/L, neutrophils 0.8 × 10^9^/L, platelets 40 × 10^9^/L and total IgG < 3.2 g/L. Repeat bone marrow trephine revealed minor residual CLL only (5%–10%). She received valaciclovir only as supportive therapy.

Serial ophthalmic examinations demonstrated an enlarging macular lesion with mild vitritis consistent with retinochoroiditis (retinal photograph, Figure 2 without satellite lesions or evidence of pre‐existing retinal scarring. Spectral‐domain optical coherence tomography Figure 1 revealed full‐thickness retinal necrosis with hyper‐reflectivity and disorganisation of retinal layers. Polymerase chain reaction (PCR) of aqueous humour demonstrated the presence of *Toxoplasma gondii*, consistent with active ocular toxoplasmosis. *T. gondii* IgG but no IgM antibodies were detected both pre‐treatment and 4 weeks from the onset of ocular symptoms. Computed tomography imaging of the brain was normal, and serum *T. gondii* PCR was negative.

*T. gondii* is a common infectious cause of retinochoroiditis. Both acquired ocular toxoplasmosis and reactivation of previous infection may be observed in immune‐competent individuals. Ocular toxoplasmosis has also been described in relapsed/refractory CLL, including those treated with continuous venetoclax. Prior fludarabine exposure has been cited as a risk factor for opportunistic infection. However, reports of toxoplasmosis during frontline CLL therapy are limited and, to our knowledge, have not included the Ven–O combination.

The patient completed systemic and intra‐vitreal clindamycin with topical prednisolone drops, noting gradual improvement of her visual acuity to 6/36 and slow healing of the macular lesion on serial examination. Given the quantitative immune dysfunction observed and evidence of past *T. gondii* infection, opportunistic reactivation of toxoplasmosis was considered. However, the absence of pre‐existing toxoplasmosis retinal scar or bilateral ocular involvement argued against infection reactivation. Absence of infection dissemination, including no central nervous system involvement as seen with acquired immune deficiency syndromes such as HIV, also argue against infection occurring as a function of immune compromise. Nonetheless, our patient remains on suppressive antibiotic therapy during Ven–O therapy and 4‐weekly prophylactic intravenous immunoglobulin.

This case highlights the complexities of infection diagnoses and management in the context of frontline novel therapies in CLL. Appropriate supportive treatments remain uncertain.

**FIGURE 1:**
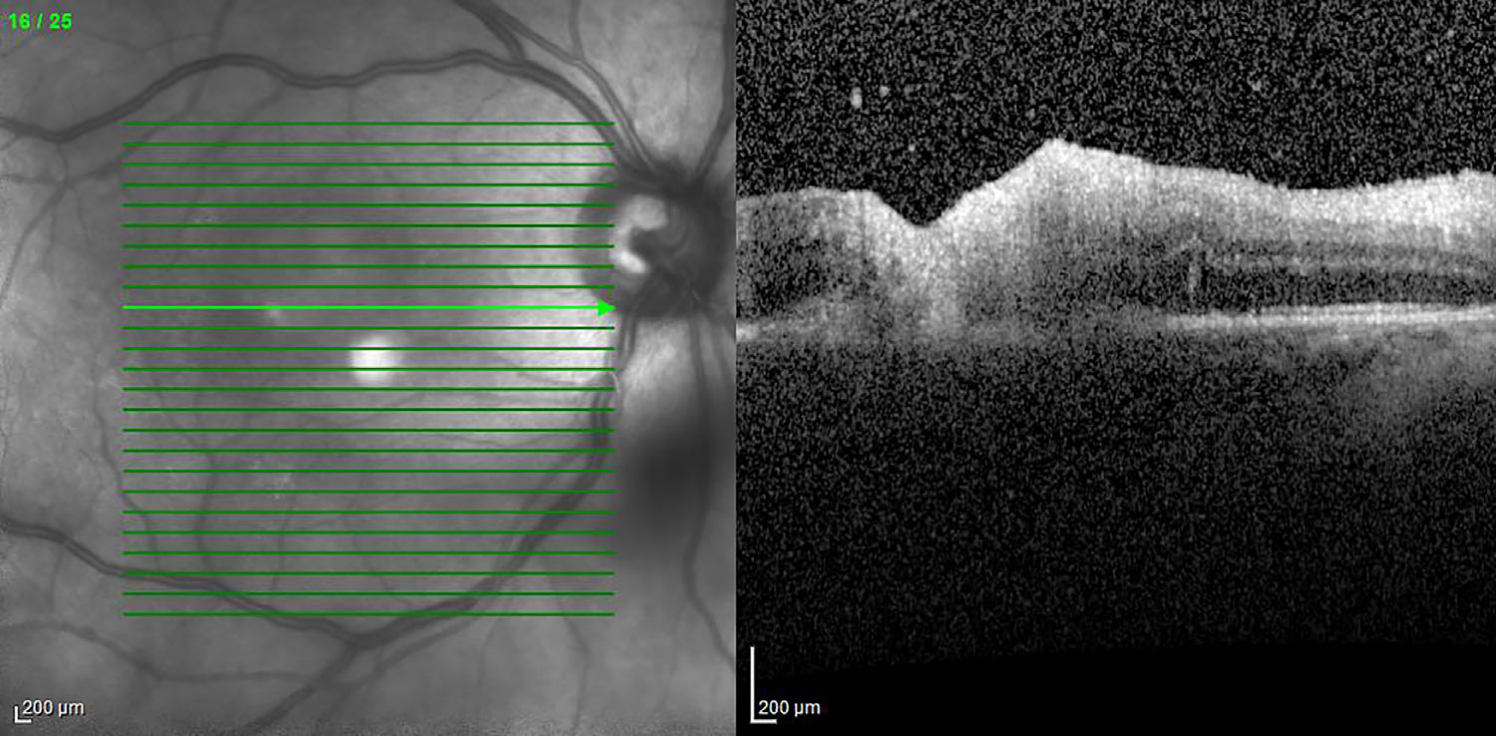
SD‐OCT

**FIGURE 2:**
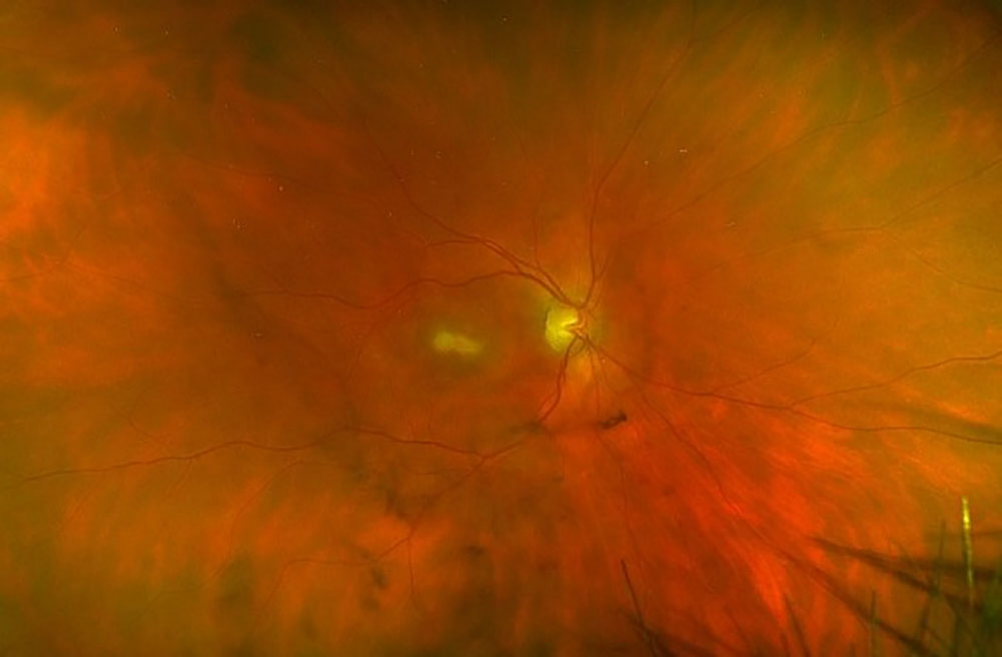
Retinal photograph

## AUTHOR CONTRIBUTIONS

Rory Bennett, Lyndell L. Lim, Constantine S. Tam interpreted the case information and wrote the manuscript.

## CONFLICT OF INTEREST STATEMENT

CST: Honoraria for AbbVie, AstraZeneca, Janssen, BeiGene and LOXO. Research funding from AbbVie, Janssen and BeiGene

## PATIENT CONSENT STATEMENT

Documented verbal consent obtained from the patient prior to preparation of manuscript.

